# Chronic Pain Treatment and Digital Health Era-An Opinion

**DOI:** 10.3389/fpubh.2021.779328

**Published:** 2021-12-10

**Authors:** V. Rejula, J. Anitha, R. V. Belfin, J. Dinesh Peter

**Affiliations:** Department of Computer Science and Engineering, Karunya Institute of Technology and Sciences, Coimbatore, India

**Keywords:** chronic pain, health innovation, artificial intelligence, eHealth, digital health, virtual care, digital biomarkers, quality of life

## 1. Introduction

Pain is acknowledged as the body alarm system that reminds us of the environmental threat, muscle damage or the presence of some disease condition (1, 2). The pain is directly related to negative emotions (3). The stimulation of primary sensory neurons and specialized transduction machinery in their peripheral terminals is thought to be the physiological cause of pain. There are two sorts of pain based on the continuous pain period, namely acute and chronic (4).

Chronic pain is defined as pain that lasts for more than 3 months (5) and it is an significant health issue. Low back pain is one of the disease that wreaks more havoc on people's lives (6–8). Other leading causes and conditions of chronic pain are Rheumatoid arthritis (9–11), Shoulder Pain (8), Headache disorders (12, 13), Cancer (14, 15), Fibromyalgia (16, 17), Cervical and Thoracic Pain (18).

Depression (19, 20), anxiety (21), sleep problems (22), fatigue/lack of energy (23), and neurocognitive abnormalities (24) are all common comorbidities of chronic pain. These comorbidities reduce the patient's quality of life, create lost workdays, and make it difficult to maintain a healthy social life on their own (25, 26).

**Figure 1:**
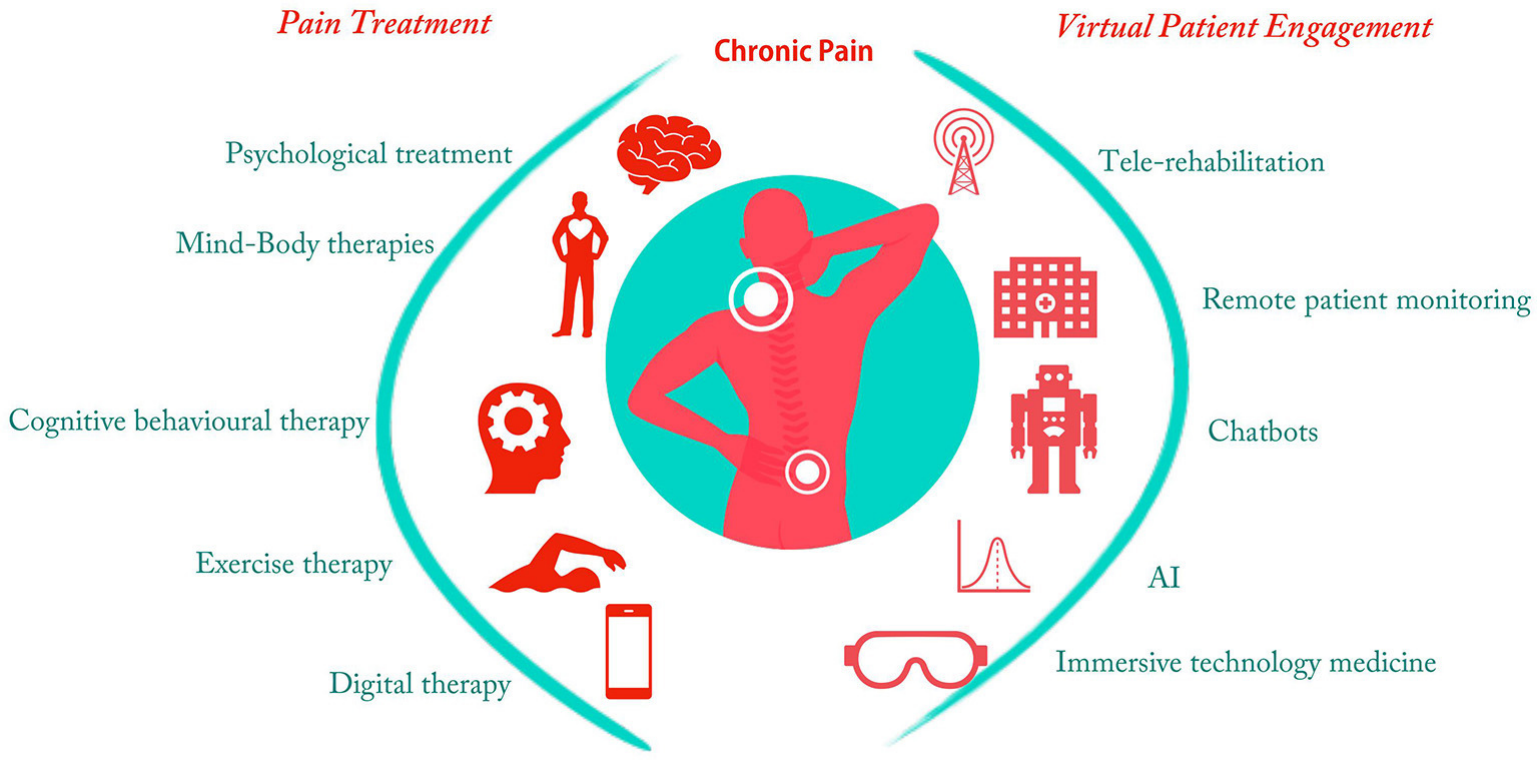
Chronic pain treatment and digital patient engagement methods.

The relevance of chronic pain research along with the techniques and problems of diagnosing chronic pain, various treatment approaches, and current digital patient interaction tools has been discussed in this article. The summary of chronic pain treatment and digital patient engagement methods has been given in Figure 1. Finally, the benefits of digital health approaches during the COVID 19 pandemic and post-pandemic era are presented.

## 2. Pain Identification Methods

There are two main aspects to pain: intensity (magnitude) and unpleasantness (effect) (27). In terms of characteristics such as duration, frequency, location, and severity, reporting the pain is essential (28). The measurement of pain can be divided into three broad classifications. “Self-report” (29) is the primary way for pain measurement. There are various scales such as Verbal Rating Scales (VRS) (30), Numerical rating scales (NRS) (31), Visual Analog Scales (VAS) (32), Smiley- based Wong-Baker Scale (WBS) (33), Faces Pain Scales-Revised (FPS-R) (34) are used to get the self-reports from the chronic pain patients (35–38). The second way of measuring pain is the “Observe behavior and infer” method (39). This method has various scales like Neonatal Infant Pain Scale (NIPS) (40), Crying Requires Increased Vital Signs Expression Sleeplessness (CRIES) (41), Face, Legs, Activity, Cry, Consolability (FLACC) (41) for Infants and Toddlers, then Pain Assessment Checklist for Seniors with Limited Ability to Communicate (PACSLAC) (42), DOLOPLUS2 (43), Pain Assessment in Advanced Dementia Scale (PAINAD) (44) for elderly with dementia and Behavioral Pain Scale (BPS) (45), Critical Care Pain Observation Tool (CPOT) (46), Nonverbal Pain Scale (NVPS) (47) for ill and unconscious persons. The third classification of measuring pain is “Indirect Physiology.” These methods use modalities like electroencephalogram (EEG) (48, 49), Magnetoencephalography (MEG) (49, 50), Positron emission tomography (PET) (7) and functional MRI (fMRI) (2, 51, 52) to measure pain using the bio-markers. Having a variety of pain measurement methods does not mean that measuring pain is simple. There are many challenges in measuring pain accurately when we use self-reports and observe behavior and infer. The following section provides the challenges in measuring pain.

### 2.1. Challenges of Measuring Pain

The “self-report” and “Observe behavior and infer” report changes depending on mood, environment, and cognition. Even after the patients have been trained, the sensitivity, robustness, and reliability of the measurements obtained by self-report are quite poor. A baby, a demented old person, or an anesthetized or coma patient cannot self-report pain. One issue with observer rating is that not everyone exhibits pain reactions. A range of behavioral and physiological changes can be used to quantify responses to noxious stimuli. Lack of signals during noxious stimulation does not always imply the absence of pain. It's critical to have measurements that can be translated into pain treatment. Hence, there is a need for a bio-marker (35).

### 2.2. Digital Bio-Markers

A bio-marker is a feature that may be tested and assessed quantitatively as an indicator of a normal biological process, a pathological process, or the pharmacological (and non-pharmacological) response to a therapeutic treatment (53). Food and Drug Administration -National Institutes of Health (FDA-NIH) (54) working group classifies bio-markers into four types according to the development of drugs and biologic:

- **Diagnostic bio-markers** -To identify and validate the presence of pain.
- **Prognostic bio-markers** -To recognize the likelihood of a clinical event, disease recurrence, or progression in patients with disease of interest.
- **Predictive bio-markers** -To identify individuals who are more likely to have the bio-marker than those who do not.
- **Pharmacodynamic bio-markers** -To show that a biological response occurs in an individual exposed to a medical product.

## 3. Chronic Pain Treatment

Chronic pain is treated in a variety of ways by healthcare practitioners. The treatment plan is determined by the type of pain, the cause (if known), and other factors that differ from person to person. Medication, lifestyle modifications, and treatments are all used in the best treatment programmes.

### 3.1. Psychological Treatment

Education is an essential element of psychological pain treatment (55) since it teaches patients how to handle a problematic condition. Talk therapy, Relaxation training, Stress management and pain coping skills training are the most frequent psychological treatments. You can obtain the assistance and counselling of a psychiatrist or psychologist through talk therapy. Cognitive therapy helps to alter the facts, ideas, and attitudes to change the amount of pain you feel. Relaxation training teaches you how to achieve a physiological state of profound relaxation that help you relieve pain. You may discover how your ideas impact your stress level and how to build a healthy response to difficult situations via stress management. You may learn how to adjust your life to the pain and have fun again through pain coping skills training (56).

### 3.2. Mind-Body Therapies

Several researchers on the use of mind-body therapy to treat persistent lower back pain have found that it is effective in reducing pain intensity, tension, and anxiety while also increasing aerobic ability. Mind-body treatments may encourage whole-body healing and include a fitness component to increase the brain's connection with physical function. They are often considered part of supplementary alternative or integrative medicine, which provides movement and energy-based therapies. Patients with persistent lower back pain benefit from integrating complementary alternative mind-body treatments in their therapy, according to a variety of medical and healthcare professionals (e.g., primary care, spine surgeons, pain management). Holistic care is sometimes coupled with traditional medical therapy (57).

### 3.3. Cognitive Behavioral Therapy

Cognitive Behavioral Therapy (CBT) combines psychotherapy (talk therapy) with behavior therapy to help individuals better comprehend life's difficulties, retrain their attitudes, ideas, and perceptions, and develop problem-solving skills. A CBT therapist guides the person through goal-oriented therapy to learn how to manage stress, anxiety, depression, and sleep issues (58).

### 3.4. Exercise Therapy

When you're dealing with chronic pain or an injury, exercise therapy is the best option. It can help you feel and move better by making you stronger. Physical therapists are skilled in both treating and preventing pain. They will search for areas of weakness or stiffness that might be contributing to the tension in the painful regions. They'll also prescribe activities to help you move better and relieve discomfort in certain places (59).

### 3.5. Digital Therapy

The use of software, mobile applications, sensors, and other digital approaches as therapeutic interventions to address various medical problems is known as digital therapy. Digital therapies refer to all of these devices and methods together. In essence, digital treatment is not dissimilar to traditional face-to-face therapeutic procedures. The cognitive behavioral therapy technique is used in the majority of digital therapy practices. It focuses on the notion of providing frequent counseling to patients to improve their behavioral and lifestyle habits (60).

## 4. Chronic Pain Virtual Patient Engagement

Many people believe that digital communication channels promote obsessive, compulsive social media illness in individuals. They are currently the effective means of contact between patients and medical professionals. We had no idea they'd become such a robust virtual tool for patient involvement. In these challenging days of the COVID-19, digital platforms assist in reaching out to patients regardless of location and save millions of lives.

### 4.1. Telerehabilitation

Telerehabilitation for pain treatment makes use of communication technologies to overcome geographical limitations. Access to such technology was indispensable during the COVID'19, and it was especially beneficial for individuals with chronic pain problems and unable to travel. Evaluating and treating such illnesses need a whole-health strategy that personalizes treatment choices and uses a biopsychosocial method to offer care. The care goals are the same as they are in a face-to-face encounter between a patient and a clinician. With proper consideration for staging a pain examination, a systematic approach to the physical visit, and the use of established clinical measures, telerehabilitation can be successfully applied in pain treatment (61).

### 4.2. Remote Patient Monitoring

RPM (remote patient monitoring) is a critical aspect of medical treatment in the future. When the objective of patient treatment is to enhance the quality of life, doctors must track how patients are doing between the appointments. This method of monitoring patients is especially crucial when dealing with chronic diseases like pain. The technologies like cloud computing, smartphone apps, sensors and wearables paved the way to improve this field (62). These software's collect, track, evaluate, and manage pain related data to improve the quality of life of a pain patient (54).

### 4.3. Chatbots

Today, algorithms-driven communications done through intelligent devices aim to treat hurts like substance use disorders, combat traumas, chronic pain and a worldwide pandemic. Conversational AI bots (chatbots) is a way to bridge the gap between the people and the knowledge they need and assisting them in completing tasks more quickly, all while utilizing existing tools. Conversational AI's future lies in incorporating it more into the care process across the whole care journey, whether it's assisting with you for appointments or just assisting you in living your best life (63, 64).

### 4.4. AI

The use of artificial intelligence in healthcare is growing, particularly in diagnosis and therapy management. AI applications in healthcare have recently made enormous impacts throughout medical services, igniting a debate about whether AI physicians would eventually displace human doctors (65). Experts, on the other hand, feel that human physicians will not be replaced by robots anytime soon. Artificial intelligence in healthcare, on the other hand, can assist doctors in making better clinical decisions or potentially replace human judgement in certain sectors of the industry.

Recent research used artificial intelligence, or machine learning algorithms, to physiological information from individuals with chronic pain, including respiration rate, oxygen levels, pulse rate, body temperature, blood pressure, and so on. The investigators' technique outperformed baseline models in determining subjective pain levels and differentiating between pain changes and irregular pain fluctuations (66).

### 4.5. Immersive Technology Medicine

A virtual reality (VR)-based digital therapy for persons with chronic pain has shown promising results in a clinical study (67), helping patients control their fear of movement, which can limit their activity and slow healing. People with chronic pain can utilize a virtual reality headset to lead them through a sequence of cognitive behavioral therapy (CBT) workouts to give them confidence in their fear of movement, commonly known as kinesiophobia (68).

## 5. Discussion

The techniques for identifying, treating, and managing chronic pain had been discussed in the preceding section. People nowadays utilize the internet and other online resources to learn about the symptoms, treatments, and comorbidities of any ailments they may be suffering. For any disease, taking the medication without first visiting a doctor is not recommended. However, the utilization of digital health data can aid medical professionals, and scientists make better informed and effective decisions.

As mentioned in previous sections, chronic pain can be self-managed by adequate patient education, lifestyle therapies such as exercise, and weight loss activities. There are various hurdles to sufficient treatment in certain underdeveloped countries, such as geography, a lack of physicians, expense, inconvenience, impairment, or, more recently, COVID'19 limitations. However, given the widespread use of computers, intelligent applications, and the world wide web, digital health methods provide the possibility of delivering lifestyle therapies to individuals with chronic pain remotely and assisting them in self-management.

As people go about their regular lives, cell phones can collect digital traces. The behavioral characteristics of a person may be derived from this raw digital data. This digital data gives us the number of unique goals visited. For example, the average level of ambient noise detected during the night, and the sleep quality data offer information on the behavior patterns of a particular individual in a real-world scenario. Combining smartphone and wearable sensors may enable passive and remote real-time monitoring of changing symptoms by capturing behavioral and physiological changes during pain. Applying machine learning classification to smartphone data and other sensor data can help us classify chronic pain patients from the controls.

In healthcare, AI has a variety of effects. In most cases, AI in healthcare uses a web data set to give doctors and specialists access to a large number of diagnostic tools. Because doctors are extremely skilled in their area and keep up with current research, AI technology in healthcare creates a much faster outcome that can be matched with their clinical expertise.

The possibilities for AI applications in healthcare are vast, ranging from emergency rooms to general care to home care. Artificial intelligence can be used in healthcare to automate patient evaluation and minimise assessor bias. It can assess patient risk, analyse sickness (for example, by decoding ECG findings and X-ray images), choose the best therapy based on a patient's clinical history and clinical trial results, track disease and detect early warning signals of worsening.

A biomarker is a biological indicator of an ailment or disease's presence. Biomarkers assist doctors in assessing and diagnosing patients as well as monitoring their therapeutic efficacy. Body temperature for fever, blood pressure for hypertension, and cholesterol levels for heart problems are examples of biomarkers. Doctors can provide target treatment by identifying and using pain biomarkers in conjunction with digital health technology. There are some drawbacks to using biomarkers for pain. Some pain biomarkers (35, 52, 69–72) are stronger predictors in males, whereas others are better predictors of pain in women concerning gene expression. CNTN1 has been related to chronic pain in women, whereas LY9 (lymphocyte antigen 9) and MFAP3 have been connected to PTSD in males. We will have higher precision when we tailor the health care application by gender since men and women have distinct biomarkers for pain in terms of gene expression. Biomarkers for pain can be identified in brain signal markers also.

Currently, 81 percent of the world's population owns a smartphone, which opens up new possibilities for remote health monitoring. The fast growth of computing technologies like mobile apps, cloud computing, blockchain, big data analytics, AI, AR & VR helps build more intelligent applications in this domain. Clinical trials and guidelines often recommend pharmacotherapy, psychotherapy, integrative therapies, and invasive procedures as part of a personalized multi-modal, multidisciplinary treatment strategy.

Incorporating the doctors with customized multi-modal treatment strategies, historical health data, advanced software technologies, and biomarkers can help the pain patients' improve their lives. The availability of huge data to train prediction algorithms, which assist (rather than replace) human doctors, stimulate curiosity-based thinking, enable cooperation, and reduce routine chores, thereby enhancing patient care, will push the application of AI in healthcare. Digital health solutions that are data-driven have the potential to change the health care domain. Suppose these technologies could be sustainably given at scale, they could provide equal access to expert-level treatment to everyone, everywhere, decreasing the worldwide health and wellness gap.

## Author Contributions

VR, JA, and RVB devised the work, the main conceptual ideas and the proof outline. VR and JA worked out almost all of the technical details. VR, RVB, and JDP worked on the manuscript. All authors contributed to the article and approved the submitted version.

## Conflict of Interest

The authors declare that the research was conducted in the absence of any commercial or financial relationships that could be construed as a potential conflict of interest.

## Publisher's Note

All claims expressed in this article are solely those of the authors and do not necessarily represent those of their affiliated organizations, or those of the publisher, the editors and the reviewers. Any product that may be evaluated in this article, or claim that may be made by its manufacturer, is not guaranteed or endorsed by the publisher.
